# Sequence variation in *Plasmodium falciparum* Histidine Rich Proteins 2 and 3 in Indian isolates: Implications for Malaria Rapid Diagnostic Test Performance

**DOI:** 10.1038/s41598-017-01506-9

**Published:** 2017-05-02

**Authors:** Praveen Kumar Bharti, Himanshu Singh Chandel, Sri Krishna, Shrikant Nema, Amreen Ahmad, Venkatachalam Udhayakumar, Neeru Singh

**Affiliations:** 1 National Institute for Research in Tribal Health (NIRTH), Garha, Jabalpur, 482003 India; 2 Malaria Branch, Division of Parasitic Diseases and Malaria, Center for Global Health, Centers for Disease Control and Prevention, Atlanta, Georgia 30329 USA

## Abstract

Commercial malaria rapid diagnostic tests (RDTs) detect *P. falciparum* histidine rich protein 2 (*Pf*HRP2) and cross react with *Pf*HRP3, a structural homologue. Here, we analysed natural variations in *Pf*HRP2 and *Pf*HRP3 sequences from Indian isolates and correlated these variations with RDT reactivity. A total 1392 *P. falciparum* positive samples collected from eight endemic states were PCR amplified for *Pfhrp2* and *Pfhrp3* genes and were sequenced. The deduced protein sequences were analysed for repeat variations and correlated with RDT reactivity. Out of 1392 PCR amplified samples, a single sample was *Pfhrp2* negative and two samples were *Pfhrp3* negative. Complete *Pfhrp*2 and *Pfhrp*3 sequences were obtained for 769 samples and 750 samples, respectively. A total of 16 distinct repeat motifs were observed for *Pfhrp2* and 11 for *Pfhrp3*, including some new repeat types. No correlation was found between variations in the size of *Pfhrp2* repeat types 2 and 7, nor between any combinations of repeat motifs, and performance of a commercial RDT at low parasite densities. The findings suggest that sequence diversity in *Pfhrp2* and *Pfhrp3* genes in Indian isolates is not likely to negatively influence performance of currently used *Pf*HRP2 RDTs.

## Introduction

Malaria is a major public health problem in India. In South Asia, India alone accounted for 80% of malaria cases and 78% of malaria deaths in 2015^1^. In India, malaria prevalence varies from state to state and the highest burden of disease mostly occurs in regions with large tribal populations^2^. Recent estimates have shown that eight out of 35 states and union territories have contributed to 80% of the total malaria cases, 85% of *Plasmodium falciparum* cases and 70% of deaths due to malaria^3^. Early diagnosis, coupled with effective treatment, is a central strategy for malaria control. Diagnosis based on microscopic examination of blood smears remains the standard national policy for case management in India. As microscopic diagnosis is not readily available in some parts of the country, especially in remote and hard to reach areas, malaria rapid diagnostic tests (RDTs) are recommended for diagnosis and treatment^4^. The availability of RDTs and the scale of their use in India have rapidly increased in recent years (about 8.5 million in 2007 to 14.5 million in 2014)^1^. The global use of RDTs in 2014 has been reported to be 314 million with 62% of the total being *P. falciparum* specific tests and the remaining 38%, combination tests that can detect more than one species^1^.

A majority of commercially available malaria RDTs target the *P. falciparum* specific histidine rich protein 2 (*Pf*HRP2) as it is a highly stable and specific biomarker. This protein is known for the abundance of multiple histidine and alanine repeats (at least 20 different repeats of mostly three, six or nine amino acids) that vary in size, frequency and composition in different parasite isolates. It has been reported that this protein typically starts with the type 1 repeat (AHHAHHVAD) followed by varying combinations of other repeats and ending with the type 12 repeat (AHHAAAHHEAATH). It is also well known that another antigenically similar protein, *Pf*HRP3, which shares some of the same amino acid repeats present in *Pf*HRP2 (types 1, 2, 4, and 7 among others), also contributes to reactivity of *Pf*HRP2 based RDTs. It is assumed that antibodies utilized in commercial RDTs most likely recognize some of these repeats but actual specificity is not well described. As far as the functional relevance of these amino acid repeats in *Pf*HRP2 for the performance characteristics of RDTs is concerned, no clear correlation between the number of repeats, size and other variations has been found^5^. However, by analysing a limited number of parasite isolate sequences with their corresponding RDT reactivity patterns at various densities, Baker *et al*. reported that the combined number of type 2 (AHHAHHAD) x type 7 (AHHAAD) repeats (cut off <43) could predict negative reactivity when parasite density is <250 parasites/µl^6^. Although this prediction was not confirmed in a subsequent study that included a large sample size (*Pf*HRP2 in 458 isolates from 38 countries), the possibility remains that some of the variation in this protein may impact the performance of RDTs, especially when the parasite density is <200 parasites/µl^5^.

Complicating the performance of *Pf*HRP2 based RDTs is the natural deletion of *Pfhrp2* genes in parasite populations in some geographical regions. A substantial proportion of parasite isolates with both *Pfhrp2* and *Pfhrp3* gene deletions, have been reported, especially in the Amazonian region of South American countries and recently in some African countries^7–9^. As these parasites will escape detection by *Pf*HRP2 based RDTs and may be selected to expand due to routine use of RDTs, there is a recommendation from WHO to conduct surveillance to detect such parasites in areas where *Pf*HRP2 RDTs are commonly used. Recently, we reported that among 1521 microscopically confirmed *P. falciparum* parasites screened from eight malaria endemic states, 50 isolates were not detected by RDTs, 36 of them had deleted *Pfhrp2* and 27 of them had deleted *Pfhrp3* ^10^. Here, we describe the natural variations in the *Pfhrp2* and *Pfhrp3* genes from all samples that were successfully sequenced using isolates obtained from the same study in India and discuss their relevance for RDT reactivity in India.

## Results

### Sequence variation in *Pfhrp2*

Out of 1392 samples, 1391 samples were amplified for *Pfhrp2* and one sample was negative. The quality of DNA was further analysed for this negative sample by amplifying the *msp1*, *msp2*, and *glurp* marker genes as recommended for determination of DNA quality^11, 12^. All three marker genes amplified in this sample and a repeat amplification for the *Pfhrp2* gene failed, thereby confirming a *Pfhrp2* deletion. However, this sample was positive for the *Pfhrp3* gene.

The PCR products of 1391 samples were sequenced and 55% (769/1391) yielded good quality sequence data (Table 1). A total of 250 unique *Pfhrp2* sequences (KX679582-831) were identified consisting of combinations of 16 different repeats (Table 2).

**Table 1:** Showing the mean parasite density, Amino acid length, number of unique and shared variants of *hrp2* and *hrp3* genes.

CHC	Parasite Density Mean	HRP2					HRP3				
		Mean Amino acid length Mean ± SD (95%CI)	No. of seq analyzed	Number of Variants (Average)	Unique variant (%)	Shared Variants (%)	Mean Amino acid Length Mean ± SD (95%CI)	No. of seq analyzed	Number of Variants (Average)	Unique variant (%)	Shared Variants (%)
Bandhugaon, District Koraput	1853 ± 370 (1128–2577)	246.3 ± 2.2 (242.0–250.7)	101	57 (0.56)	10 (10.4)	10 (8.3)	167.3 ± 2.0 (163.4–171.1)	114	34 (0.30))	4 (5.7)	18 (12.3)
Jagannathpur, District Rayagada	1214 ± 318 (590–1837)	239.2 ± 3.8 (231.7–246.7)	24	15 (0.63)	3 (3.1)	6 (5.0)	171.1 ± 5.1 (161.2–181.1)	7	5 (0.71)^^^	2 (2.9)	3 (2.1)
Jaldega, District Simdega	2025 ± 484 (1076–2974)	247.2 ± 2.6 (242.0–252.3)	71	50 (0.70)	17 (17.7)	8 (6.7)	165.5 ± 1.4 (162.6–168.3)	65	22 (0.34)	4 (5.7)	14 (9.6)
Bano, District Simdega	5775 ± 2175 (1508–10041)	245.4 ± 2.0 (241.5–249.2)	66	48 (0.73)^^^	10 (10.4)	9 (7.5)	164.5 ± 2.2 (160.2–168.8)	8	3 (0.38)	0 (0.0)	3 (2.1)
Jagdalpur	13778 ± 2725 (8433–19123)	245.7 ± 2.2 (241.3–250.1)	111	74 (0.67)	20 (20.8)	12 (10.0)	163.6 ± 1.3^#^ (161.1–166.1)	129	41 (0.32)	15 (21.4)	17 (11.6)
Baikunthpur	1253 ± 394 (479–2026)	244.4 ± 3.8 (237.0–251.8)	5	5 (1.00)	1 (1.0)	3 (2.5)	169.3 ± 6.8 (155.9–182.7)	6	3 (0.50)	1 (1.4)	2 (1.4)
Ranapur, District Jhabua	4846 ± 949 (2986–6707)	242.2 ± 3.3 (235.6–248.8)	77	32 (0.42)^#^	5 (5.2)	11 (9.2)	174.4 ± 2.0 (170.5–178.3)	94	22 (0.23)^#^	10 (14.3)	11 (7.5)
Pushprajgarh, District Anuppur	8647 ± 1435 (5833–11462)	250.6 ± 3.7 (243.3–257.9)	52	26 (0.50)	5 (5.2)	9 (7.5)	161.3 ± 1.3^#^ (158.7–163.9)	67	21 (0.31)	2 (2.9)	14 (9.6)
Malewada, District Gadchiroli	2279 ± 415 (1465–3093)	237.2 ± 4.0 (229.4–245.1)	40	30 (0.75)^^^	4 (4.2)	10 (8.3)	166.7 ± 2.5 (161.7–171.7)	45	22 (0.49)^^^	4 (5.7)	14 (9.6)
Darekasa, District Gondia	6605 ± 811 (5013–8196)	240.1 ± 2.6 (234.9–245.2)	37	20 (0.54)	7 (7.3)	6 (5.0)	168.5 ± 3.3 (162.0–175.0)	28	15 (0.54)^^^	4 (5.7)	10 (6.8)
Bekaria, District Udaipur	3735 ± 1044 (1686–5784)	242.9 ± 3.0 (236.9–248.8)	79	32 (0.41)^#^	1 (1.0)	10 (8.3)	169.8 ± 2.3 (165.1–174.4)	79	17 (0.22)^#^	4 (5.7)	13 (8.9)
Devgadh Baria, District Dahod	1945 ± 518 (929–2961)	231.7 ± 3.9^#^ (224.0–239.4)	34	21 (0.62)	2 (2.1)	10 (8.3)	174.8 ± 2.2^^^ (170.4–179.2)	53	15 (0.28)	4 (5.7)	9 (6.2)
Lavkar, District Valsad	1273 ± 413 (463–2083)	237.5 ± 13.2 (211.5–263.5)	6	5 (0.83)	1 (1.0)	2 (1.7)	202.0 ± 5.2^^^ (191.8–212.2)	4	4 (1.00)^^^	1 (1.4)	3 (2.1)
Manu bazar, South Tripura	6540 ± 1352 (3888–9191)	235.3 ± 3.8 (227.9–242.6)	27	15 (0.56)	4 (4.2)	6 (5.0)	178.4 ± 5.6 (167.5–189.4)	14	10 (0.71)^^^	3 (4.3)	6 (4.1)
Santir bazar SouthTripura	10580 ± 1782 (7085–14075)	240.7 ± 3.9^#^(233.0–248.3)	39	26 (0.67)	6 (6.3)	8 (6.7)	171.0 ± 3.6 (163.9–178.0)	37	24 (0.65)^^^	12 (17.1)	9 (6.2)

^^^The mean is significantly higher than Overall Average; ^#^The mean is significantly lower than Overall mean; Average = average is calculated from number of variants divided by the total number of samples analysed.

**Table 2:** Types of Amino acid repeats in *Pf*HRP2 and *Pf*HRP3.

Type	Amino acid repeat sequence	*Pf*HRP2	Percentage	*Pf*HRP3	Percentage
1	AHHAHHVAD	+	98.3	+	100.0
2	AHHAHHAAD	+	100.0	+	2.9
3	AHHAHHAAY	+	93.8	−	0
4	AHH	+	31.9	+	99.9
5	AHHAHHASD	+	72.7	−	0
6	AHHATD	+	100.0	−	0
7	AHHAAD	+	100.0	+	99.9
8	AHHAAY	+	96.5	−	0
9	AAY	−	0	−	0
10	AHHAAAHHATD	+	90.1	−	0
11	AHN	−	0	−	0
12	AHHAAAHHEAATH	+	100.0	−	0
13	AHHASD	+	3.8	−	0
14	AHHAHHATD	+	6.6	−	0
15	AHHAHHAAN	−	0	+	99.7
16	AHHAAN	−	0	+	98.1
17	AHHDG	−	0	+	100.0
18	AHHDD	−	0	+	99.6
19	AHHAA	+	1.2	−	0
20	SHHDD	−	0	+	100
21	AHHAHHATY	−	0	−	0
22	AHHAHHAGD	−	0	−	0
23	ARHAAD	−	0	−	0
24	AHHTHHAAD	−	0	−	0
25	**AHHASY**	+	3	−	0
26	**AHHAHHVSD**	+	1	−	0
27	**AHHSHHAAD**	+	0.5	−	0
28	**SHHDG**	−	0	+	2.0
29	**AHHVAD**	−	0	+	3.1

**Figure 1:**
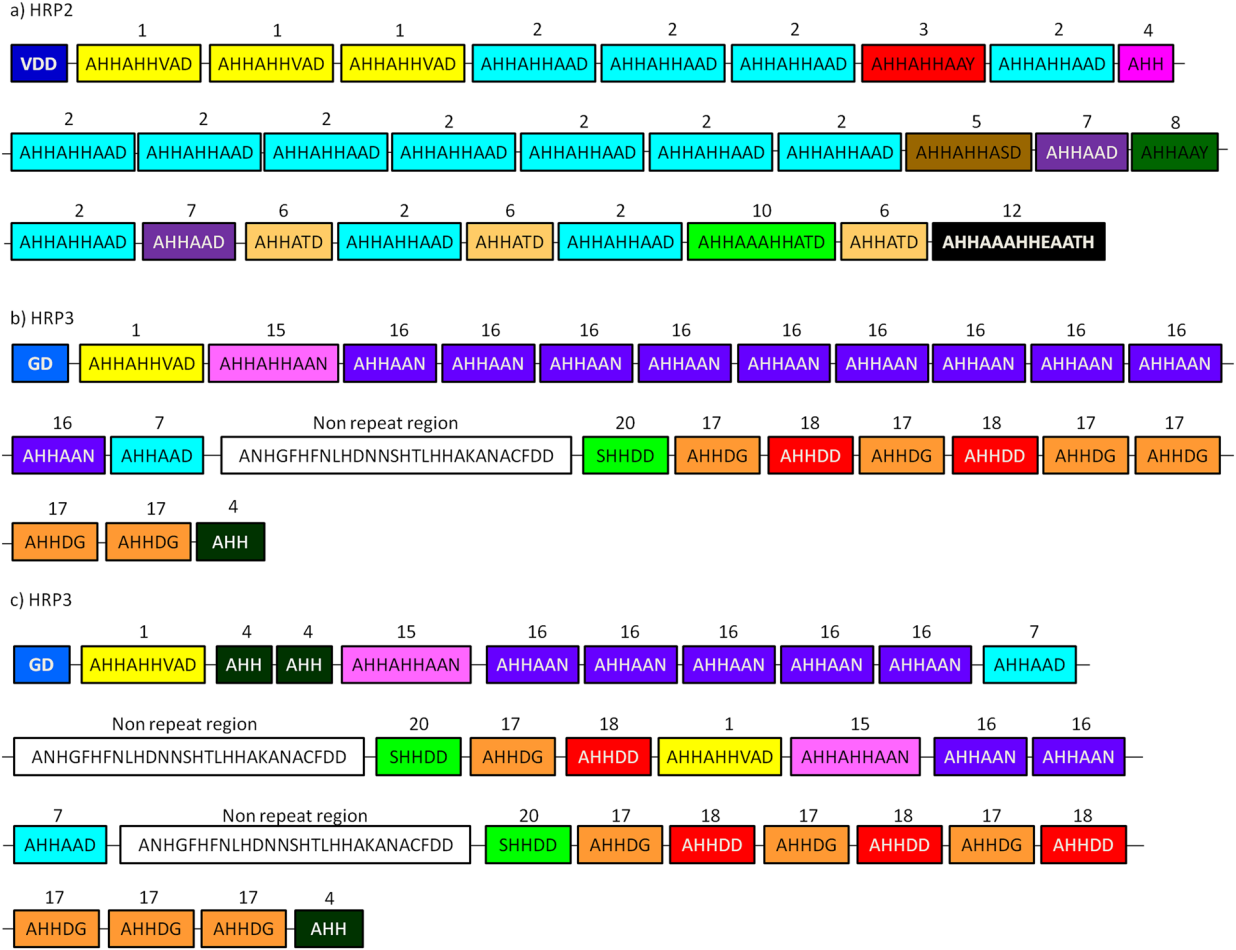
Schematic diagram showing location and arrangement of amino acid repeats in the *P. falciparum* protein among Indian isolates based on present study. In both the HRP2 and HRP3, repeat region start from the type 1 repeat (AHHAHHVAD). In HRP3 few isolates were having one non-repeat region while other were having two non-repeat region. **(a**) Showing arrangement of repeats in *Pf*HRP2, (**b**) and (**c**) showing arrangement of repeats in *Pf*HRP3.

**Figure 2:**
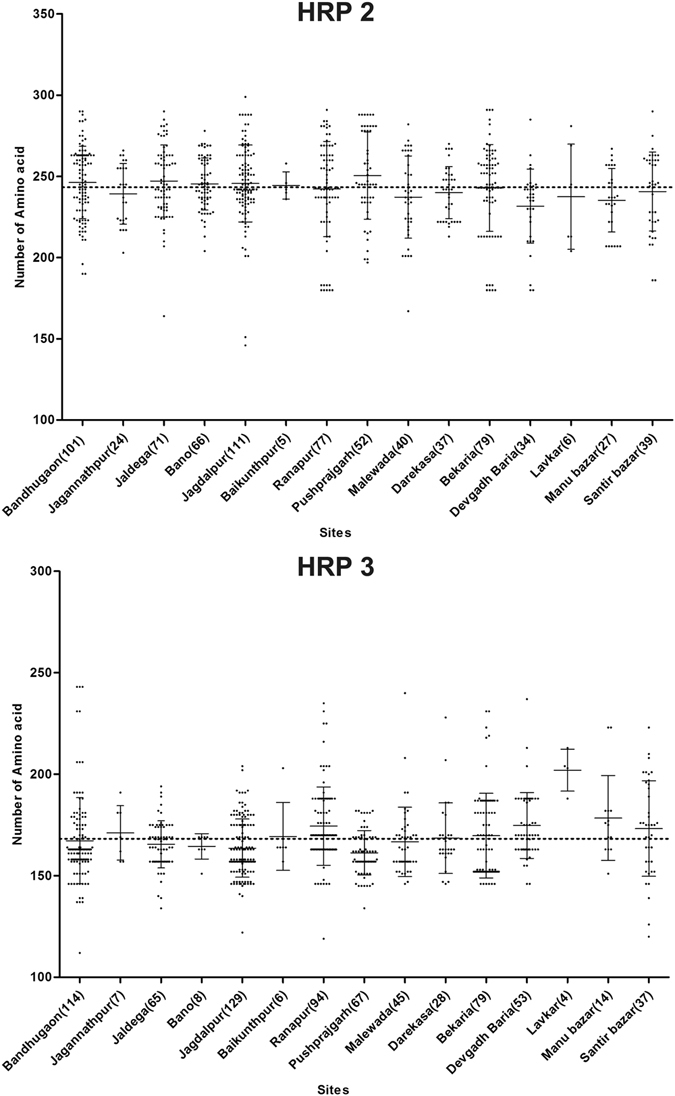
The variation in the length of amino acids in *Pf*HRP2 and *Pf*HRP3 protein from study sites. Number in brackets indicates the number of samples. The dotted line indicates the country mean. ^^^The mean is significantly higher than the country mean (p < 0.05); ^#^the mean number is significantly lower than the country mean.

The size of the repeat region of exon 2 varied from 438 to 897 bases (mean 730). The difference in the mean size of exon 2 bases for each site is reported in Table 1. The size variation was largely attributed to the variation in numbers of 27, 18 and 9 bp repeats. Based on the data from this study the common arrangement of the repeats was drawn for HRP2 and HRP3 (Fig. 1). Among the 16 different types of repeats observed in this study, 13 were reported previously in other global populations of parasites^6^. The type 25 (AHHASY), type 26 (AHHAHHVSD) and type 27 (AHHSHHAAD) repeat motifs were unique sequences found in this study (Table 2). The length of the deduced exon-2 *Pfhrp2* sequence varies from 146 to 299 aa (mean 243 aa). When compared to the country mean, the mean of amino acid length was found to be significantly lower in both Gujarat and Tripura States (p < 0.05, Fig. 2).

### Variation in the number of repeats

The total number of repeats and the number of each repeat within *Pfhrp2* varied between isolates, both within and between sites (Tables S1 and S2). Repeat types 2, 6, 7 and 12 were observed in 100% of the isolates sequenced (Table 2). Repeat types 1, 3, and 10 were observed in 90% of the samples while types 5 and 8 were found in over 80% samples. The prevalence of repeat types 13, 14, 25, 26 and 27 were found to differ between sites from 3 to 10%. Further analysis was performed to examine the differences in the number of type 2 and 7 repeats in parasite isolates from different sites (Fig. S1). The type 2 repeats are the largest in size comprising about 47% of the total repeats followed by type 7 repeats accounting for 12% (Table S3).

### Sequence variation in *Pfhrp3*

Out of 1392 samples, 1390 samples were amplified for the *Pfhrp3* gene. Two negative samples had deleted the *Pfhrp3* gene. The PCR products of 1390 samples were sequenced and 54% (750/1390) yielded good quality sequences. The size of *Pfhrp3* repeat region of exon 2 amplified by PCR ranged from 336 to 729 bp (mean 504 bp). Out of the total 750 sequences, 137 unique *Pfhrp3* sequences were found (KX679832-968). The length of the deduced *Pfhrp3* sequence encoded by the repeat region of exon-2 varies from 112 to 243 amino acids (mean 168 aa). The mean amino acid length was significantly higher in Gujarat State and significantly lower in Chhattisgarh State when compared to the country mean (p < 0.05, Table 2). Overall, the level of diversity in the *Pfhrp3*, as measured by the proportion of sequence variations, is significantly lower than the *Pfhrp2* gene (p < 0.05).

Eleven different repeats were present in *Pf*HRP3 (Table 2). Nine repeats were identical to previously reported sequences^5^ and two new repeat types were identified in this study. All the sequences started with a type 1 repeat (AHHAHHVAD) and ended with a type 4 repeat (AHH). The remaining repeat types accounted for the central part of the protein. The site and state-wide variations in repeats are summarized in (Tables S4 and S5). All isolates had at least a single stretch of 28 amino acid long non-repeat sequence. In some isolates, a second copy of the same non-repeat sequence was found (Fig. 1c). Although this non-repeat sequence is highly conserved, a limited number of non-synonymous polymorphisms were observed (Table S6). The repeat types 4, 7, 16–18 and 20 were found in 90% to 100% of isolates while the presence of other repeats varied. The number of repeats across different sites is highly variable including type 16 and type 17 repeats in parasite isolates from different sites (Fig. S1). The type 16 and 17 combined repeats are the largest in size comprising of about 50% of the total repeats followed (Table S7).

### Correlation between repeat lengths and RDT positivity

**Figure 3:**
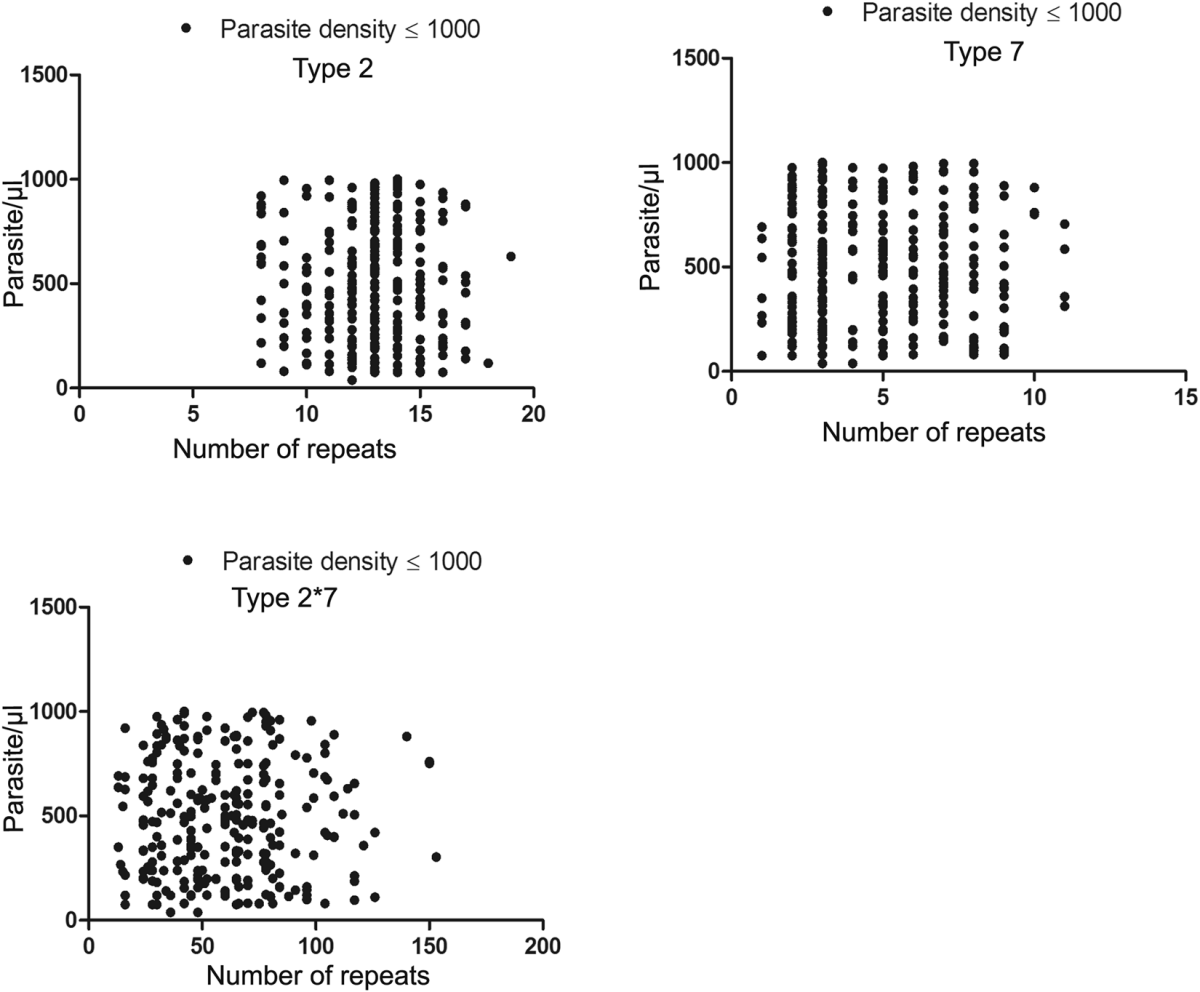
Association between parasite density and number of repeat among the samples having ≤1000 parasite per microlitre. Parasite density in Type 2, Type 7 and Type 2 × 7 repeats.

The impact of natural variations in *Pf*HRP2 repeats in field isolates on the performance of RDTs, especially in detecting low density infections in field settings, is not well understood. The relationship between number and combination of major repeat types 2 and 7 was investigated in low density infections (<1,000 parasites/$\mu$l) (Fig. 3). No direct relationship between these two type repeats and the ability to detect low density infections was observed. Indeed, there were 15 very low density infections (<200 parasites/µl) and there was no difference in the ability of an RDT to detect such infections due to variations in the size of repeats or in combinations of type 2 and 7 repeats (Fig. 3). Similarly, no relationships between type 6 and 10 repeats ‘and RDT positivity were observed (data not shown).

## Discussion

Malaria RDTs have become widely valued diagnostic tools for resource limited settings, including India, where a large burden of malaria occurs in economically disadvantaged tribal populations^13–15^. The successful implementation of evidence based treatment of malaria infections, as recommended by WHO, became feasible due to the availability of several commercial RDTs. *Pf*HRP2 based RDTs are the most commonly used RDTs in the field and demonstrate a high detection rate; however, their accuracy is variable between similar products and even between different lots^16^. The performance of RDTs in the field can be influenced by a number of factors but the impact of high degree of variation in the *Pf*HRP2 antigen sequences^17^, especially in the detection of low density infections at the threshold of detection limit (<200 parasites/µl), has been a subject of investigation^5, 6^. Our investigation is one of the largest studies reported to date that determined the natural variations in the *Pfhrp2* gene, and its paralogue *Pfhrp3*, in field isolates and examined the impact of such variation in detecting low density infections by a commonly used RDT.

This study clearly demonstrates the extensive variation in both *Pfhrp2* and *Pfhrp3* genes in Indian parasite isolates consistent with previous studies that have analysed variations in field isolates from various geographical regions, including India^5, 6, 18–20^. Genetic variation in *Pfhrp2* and *Pfhrp3* was found in all sites with different epidemiological characteristics (Table 1). Significantly higher genetic variations were observed from the states of Jharkhand and Maharashtra and significantly lower from the states of Madhya Pradesh and Rajasthan (Table S2). Although higher variation was also found in Odisha and Gujarat States, the differences were not statistically significant (p > 0.05).

As various repeats constitute the majority of amino acids in both *Pf*HRP2 and *Pf*HRP3, our analysis focused on the deduced amino acid variations, frequency of repeats and their organization. The overall size of repeat region amino acids varied considerably within and between sites. The type 2 repeats contributed to nearly half (47%) of all repeat amino acid sequences while type 7 repeats comprised 12%, this pattern is comparable to previous studies^5, 6, 18^. In the present study, the number of type 2 repeats varied from 12 to 15 and only the isolates from Madhya Pradesh showed significantly lower prevalence of type 2 repeats as compared to the country mean (p < 0.05). In contrast, the type 7 repeat was significantly higher in Madhya Pradesh compared to the country mean (Table S2). The type 1, 3, 6, 8, 10 and 12 repeats were the other most common repeats in the *Pf*HRP2 while type 4, 5, 13, 14 and 19 and three new repeat types (25, 26 and 27) varied between isolates. Overall these results are consistent with previous reports^6, 19^.

Types 1, 4, 7, 15, 16, 17, 18 and 20 repeats were the common repeats in *Pf*HRP3 as reported previously from global isolates^6^. In this study, two non-repetitive regions were found in all the states; however, they were more common among Rajasthan and Tripura isolates. Baker *et al*.^5^, reported that only limited isolates from three countries showed two non-repetitive regions. The location of the non- repetitive region varied between our study and previous reports^6^ (Fig. 1c).

We have reported previously *Pfhrp2* and *Pfhrp3* genetic deletions in the 50 samples that were RDT negative but microscopy positive^10^. It is important to note that we found one additional *Pfhrp2* deleted and two *Pfhrp3* deleted samples among the 1392 samples subjected for PCR amplification. This observation suggests that our approach, as recommended by WHO to test samples that failed RDTs after microscopic confirmation of parasitemia, did not underestimate the prevalence of *Pfhrp2* and *Pfhrp3* deletion in this study. However, it is important to point out that the only sample with *Pfhrp2* deletion was positive for *Pfhrp3* gene and this must have contributed to positive RDT detection of this sample as previously observed^21^. Similarly, the two *Pfhrp3* negative samples were positive for *Pfhrp2* gene accounting for their positive RDT detection.

As Baker *et al*. demonstrated, that if a combined length of type 2 x type 7 *Pf*HRP2 proteins was <43 repeat length, then it predicted the negative reactivity of RDTs at parasite densities <250 parasites/µl. This finding was made by testing the reactivity of 16 cultured isolates at different dilutions of *in vitro* grown parasites with a panel of RDTs and analysing the reactivity pattern using a binary regression model^6^. Although their own subsequent study involving large sample size failed to confirm the validity of their model^5^, some studies including a previous Indian study have reported some correlation between types 2 × 7 repeat lengths and RDT reactivity at low density^18, 19^. In the present study, we compared the RDT reactivity pattern of these samples with their own deduced amino acid variation, which reflects a direct correlation between RDT reactivity and variation pattern. The SD Bioline RDT used in this study was found to show a lower sensitivity at densities <1,000 parasites in previous field evaluations in India^14^. We compared the RDT reactivity pattern of all samples with a parasite density of <1,000 parasites with the repeat length of type 2 × 7 (Fig. 3). Interestingly, we did not find any correlation between length variation of these repeats and combined length of type 2 and 7. Importantly, there were several isolates with low density parasitemia (<200 parasites/µl) as well as <43 amino acids of combined repeats of type 2 × 7 but were clearly detected by the RDT^5^. Overall these results are consistent with Baker *et al*.’s subsequent conclusion that variations in length of any of repeats or type 2 × 7 combination did not show any correlation with RDT reactivity at lower parasite density (<200 parasites/µl). Based on this observation, we suggest that it will be worth comparing the variation between RDT reactivity and repeat variations in the field, rather than in *in-vitro* cultured parasite dilutions to further confirm current observations. The limitation of the study is that we could not analyze all available samples as we had to exclude samples that did not yield good quality sequences and these samples were also not attempted for further re-sequencing.

## Conclusion

This study provides the first country-wide data on genetic diversity of *P. falciparum hrp2* and *hrp3* genes. The findings confirm that both *Pfhrp2* and *Pfhrp3* had extreme variation between the type and number of the repeats. There was no correlation between length variation of the repeats type 2 and 7 with RDT positivity, even at low density parasitemia. Among the RDT positive samples, only a single sample was *Pfhrp2* deleted and two samples were *Pfhrp3* deleted. Our findings may lead to a better understanding of the *Pfhrp2* structure and how its variation contributes to the RDT positivity and in turn, also help in the generation of improved malaria RDTs.

## Material and Methods

### Study details and sample source

In a previous study, we determined the natural genetic variation of *Pfhrp2* and *Pfhrp3* genes in *Plasmodium falciparum* samples obtained from a total of sixteen sites from eight malaria endemic states in India with a low level prevalence of *Pfhrp2* and *Pfhrp3* deleted parasites^10^. These samples were used in this investigation to characterize natural variations in *Pfhrp2* and *Pfhrp3* genes. Details of individual study sites and parasitemia data are reported in Table 1. Out of 16 sites, eight exhibited high level of malaria endemicity (Annual parasite incidence >5) and 8 sites had low malaria endemicity (Annual parasite incidence <2). Out of 16 sites, *P. falciparum* positive blood samples were collected from 15 sites. The epidemiological characteristics and details of the study sites have been previously described^3, 10^.

### Ethical approval

The study protocol for patient participation and collection of blood samples for laboratory testing from participants were approved by the institutional ethics committee of National Institute for Research in Tribal Health (NIRTH), Jabalpur. Before collecting the samples, written informed consent was obtained from all study participants or from the parents/guardian of children, as per the guidelines of the Indian council of medical research. A copy of the consent form in the local language was also provided and explained to the patients or parents/guardians of children. The participation by a CDC investigator was approved under a non-research determination by the Center for Global Health, CDC, Atlanta.

### Sample Selection and genetic variation study

Out of these 1521 samples, RDT test results confirmed 1471 samples were positive for *P. falciparum* and 50 samples were negative^10^. Out of 1471 microscopically and RDT positive samples, there were 1392 samples were available for present study. All the 1392 samples were amplified for *hrp2* and *hrp3* genes by using the protocol described earlier^10^. Nucleotide and amino acid sequences were submitted in the NCBI (Genbank accession number KX679582-KX679968).

### Statistical analysis

Differences in the presence/absence of amino acid repeats in the isolates from different sites/ states were assessed by Chi square tests and differences in the mean number of amino acid repeats between the states were assessed using the Kruskall-Wallis test (H test) for each type of repeat. P values < 0.05 were interpreted as indicating statistically significant difference.

## Electronic supplementary material

supplimentry table and figure
