# Supplementary material for: Sequence variation in Plasmodium falciparum Histidine Rich Proteins 2 and 3 in Indian isolates: Implications for Malaria Rapid Diagnostic Test Performance
Source: Sci Rep. 2017 May 2;7:1308. doi: 10.1038/s41598-017-01506-9 (PMC5430971; doi:10.1038/s41598-017-01506-9)
Supplement: Supplementary file 1 — supplimentry table and figure [file 41598_2017_1506_MOESM1_ESM.pdf]

Sequence variation in *Plasmodium falciparum* Histidine Rich Proteins 2 and 3 in Indian isolates: Implications for Malaria Rapid Diagnostic Test Performance

Praveen Kumar Bharti[1], Himanshu Singh Chandel[1], Sri Krishna[1], Shrikant Nema[1], Amreen Ahmad[1], Venkatachalam Udhayakumar[2] and Neeru Singh[1*]

[1]National Institute for Research in Tribal Health (NIRTH), Garha, Jabalpur, 482003 India.

[2]Malaria Branch, Division of Parasitic Diseases and Malaria, Center for Global Health, Centers for Disease Control and Prevention, Atlanta, Georgia, 30329 USA.

[*]Corresponding author:

E-mail: neeru.singh@gmail.com

Supplementary information:

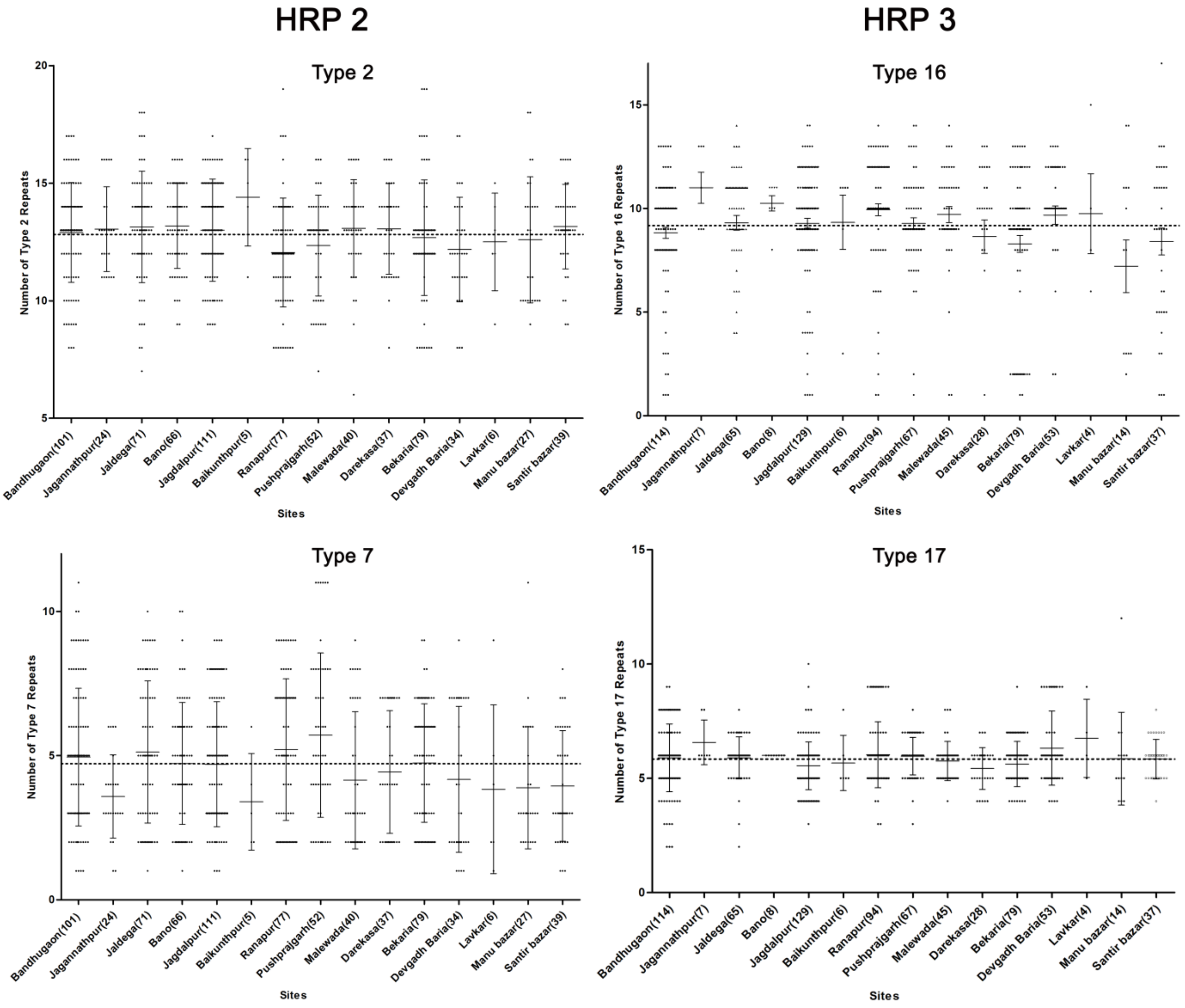

Figure 1: The mean number of different repeat types present in *Pf*HRP2 and *Pf*HRP3. Showing the average number of times type 2 and type 7 present in *Pf*HRP2 and repeat type 16 and 17 present in *Pf*HRP3 samples isolated from study sites.

Table 1: The percentage of various types of *Pf*HRP2 repeats present in eight different states of India.

State	CHC	n	Type1	Type2	Type3	Type4	Type5	Type6	Type7	Type8	Type10	Type12	Type13	Type14	Type19	Type25	Type26	Type27
Orissa	Bandhugaon, District Koraput	101	100.0	100.0	89.1	28.7	65.3	100.0	100.0	97.0	82.2	100.0	3.0	8.9	3.0	4.0	0.0	1.0
	Jagannathpur, District Rayagada	24	100.0	100.0	100.0	37.5	70.8	100.0	100.0	79.2	79.2	100.0	8.3	12.5	0.0	0.0	0.0	0.0
Jharkhand	Jaldega, District Simdega	71	100.0	100.0	88.7	33.8	63.4	100.0	100.0	98.6	87.3	100.0	4.2	4.2	1.4	0.0	0.0	1.4
	Bano, District Simdega	66	100.0	100.0	86.4	34.8	68.2	100.0	100.0	98.5	87.9	100.0	3.0	4.5	0.0	0.0	0.0	0.0
Chhattisgarh	Jagdalpur	111	99.1	100.0	94.6	28.8	61.3	100.0	100.0	97.3	88.3	100.0	1.8	9.9	1.8	0.9	0.0	0.9
	Baikunthpur	5	100.0	100.0	100.0	40.0	80.0	100.0	100.0	100.0	100.0	100.0	0.0	0.0	0.0	0.0	0.0	0.0
Madhya Pradesh	Ranapur, District Jhabua	77	100.0	100.0	97.4	36.4	89.6	100.0	100.0	93.5	100.0	100.0	1.3	9.1	2.6	0.0	5.2	0.0
	Pushprajgarh, District Anuppur	52	92.3	100.0	96.2	36.5	80.8	100.0	100.0	100.0	92.3	100.0	0.0	19.2	0.0	0.0	0.0	0.0
Maharashtra	Malewada, District Gadchiroli	40	90.0	100.0	85.0	37.5	65.0	100.0	100.0	92.5	90.0	100.0	0.0	5.0	2.5	2.5	0.0	2.5
	Darekasa ,District Gondia	37	100.0	100.0	100.0	16.2	78.4	100.0	100.0	100.0	100.0	100.0	0.0	0.0	0.0	27.0	0.0	0.0
Rajasthan	Bekaria, District Udaipur	79	100.0	100.0	96.2	38.0	81.0	100.0	100.0	93.7	94.9	100.0	5.1	0.0	0.0	0.0	3.8	0.0
Gujarat	Devgadh Baria, District Dahod	34	97.1	100.0	97.1	58.8	82.4	100.0	100.0	97.1	88.2	100.0	14.7	0.0	0.0	0.0	2.9	0.0
	Lavkar ,District Valsad	6	100.0	100.0	100.0	16.7	83.3	100.0	100.0	100.0	100.0	100.0	16.7	16.7	0.0	0.0	0.0	0.0
Tripura	Manu bazar, South Tripura	27	100.0	100.0	100.0	11.1	92.6	100.0	100.0	100.0	96.3	100.0	7.4	0.0	0.0	7.4	0.0	0.0
	Santir bazar, South Tripura	39	92.3	100.0	100.0	10.3	66.7	100.0	100.0	100.0	84.6	100.0	10.3	5.1	0.0	12.8	0.0	0.0
**Overall**		**769**	**98.3**	**100.0**	**93.8**	**31.9**	**72.7**	**100.0**	**100.0**	**96.5**	**90.1**	**100.0**	**3.8**	**6.6**	**1.2**	**3.0**	**1.0**	**0.5**

4 **Table 2: The mean number of different type of repeats present in *Pf*HRP2.**

CHC	n	Type1 Mean ± SD (95%CI)	Type2 Mean ± SD (95%CI)	Type3 Mean ± SD (95%CI)	Type4 Mean ± SD (95%CI)	Type5 Mean ± SD (95%CI)	Type6 Mean ± SD (95%CI)	Type7 Mean ± SD (95%CI)	Type8 Mean ± SD (95%CI)	Type10 Mean ± SD (95%CI)	Type12 Mean ± SD (95%CI)	Type13 Mean ± SD (95%CI)	Type14 Mean ± SD (95%CI)	Type19 Mean ± SD (95%CI)	Type25 Mean ± SD (95%CI)	Type26 Mean ± SD (95%CI)	Type27 Mean ± SD (95%CI)
Bandhugaon, District Koraput	101	2.5 ± 0.1 (2.3 -2.8)	12.9 ± 0.2 (12.5 -13.3)	1.2 ± 0.1 (1.1 -1.3)	0.4 ± 0.1 (0.2 -0.5)	0.8 ± 0.1 (0.6 -0.9)	3.3 ± 0.1 (3.1 -3.5)	5.0 ± 0.2 (4.5 - 5.4)	1.1 ± 0.0 (1.0 -1.2)	1.3 ± 0.1 (1.2 - 1.5)	1.0 ± 0.0	0.1 ± 0.1 (0.0 -0.2)	0.1 ± 0.0 (0.0 -0.1)	0.0 ± 0.0 (0.0 -0.1)	0.0 ± 0.0 (0.0 -0.1)	0.0	0.0
Jagannathpur, District Rayagada	24	2.8 ± 0.2 (2.4 - 3.3)	13.0 ± 0.4 (12.3 - 13.8)	1.3 ± 0.1 (1.1 - 1.5)	0.4 ± 0.1 (0.2 -0.6)	0.7 ± 0.1 (0.5 - 0.9)	3.1 ± 0.2 (2.7 - 3.5)	3.6 ± 0.3$^{\#}$ (3.0 - 4.2)	0.8 ± 0.1$^{\#}$ (0.6 - 1.0)	1.3 ± 0.2 (1.0 - 1.6)	1.0 ± 0.0	0.1 ± 0.1 (0.0 - 0.2)	0.1 ± 0.1 (0.0 - 0.3)	0.0	0.0	0.0	0.0
Jaldega, District Simdega	71	2.3 ± 0.1 (2.0 -2.6)	13.1 ± 0.3 (12.6 -13.7)	1.2 ± 0.1 (1.1 - 1.3)	0.4 ± 0.1 (0.3 - 0.6)	0.7 ± 0.1 (0.6 - 0.9)	3.1 ± 0.1 (2.8 - 3.3)	5.1 ± 0.3 (4.6 - 5.7)	1.3 ± 0.1$^{\wedge}$ (1.2 - 1.4)	1.4 ± 0.1 (1.2 - 1.5)	1.0 ± 0.0	0.1 ± 0.1 (0.0 - 0.2)	0.1 ± 0.0 (0.0 - 0.2)	0.0 ± 0.0 (0.0 - 0.0)	0.0	0.0	0.0
Bano, District Simdega	66	2.4 ± 0.1 (2.2 - 2.6)	13.2 ± 0.2 (12.7 - 13.6)	1.2 ± 0.1 (1.0 - 1.3)	0.4 ± 0.1 (0.2 - 0.5)	0.8 ± 0.1 (0.6 - 0.9)	3.2 ± 0.1 (2.9 - 3.4)	4.7 ± 0.3 (4.2 - 5.2)	1.1 ± 0.1 (1.0 - 1.2)	1.4 ± 0.1 (1.2 - 1.5)	1.0 ± 0.0	0.0 ± 0.0 (0.0 - 0.1)	0.1 ± 0.0 (0.0 - 0.2)	0.0	0.0	0.0	0.0
Jagdalpur	111	2.5 ± 0.1 (2.3 -2.7)	13.0 ± 0.2 (12.6 - 13.4)	1.3 ± 0.1 (1.2 - 1.4)	0.4 ± 0.1 (0.2 -0.5)	0.7 ± 0.1 (0.6 - 0.8)	3.2 ± 0.1 (3.0 - 3.4)	4.7 ± 0.2 (4.3 - 5.1)	1.1 ± 0.0$^{\#}$ (1.0 - 1.1)	1.4 ± 0.1 (1.3 - 1.5)	1.0 ± 0.0	0.0 ± 0.0$^{\#}$ (0.0 - 0.0)	0.1 ± 0.0 (0.0 - 0.2)	0.0 ± 0.0 (0.0 - 0.0)	0.0 ± 0.0$^{\#}$ (0.0 - 0.0)	0.0	0.0
Baikunthpur	5	1.8 ± 0.2$^{\#}$ (1.4 -2.2)	14.4 ± 0.9 (12.6 -16.2)	1.2 ± 0.2 (0.8 - 1.6)	0.6 ± 0.4 (-0.2 - 1.4)	0.8 ± 0.2 (0.4 - 1.2)	3.2 ± 0.4 (2.5 - 3.9)	3.4 ± 0.7 (1.9 - 4.9)	1.2 ± 0.2 (0.8 - 1.6)	1.4 ± 0.2 (0.9 - 1.9)	1.0 ± 0.0	0.0	0.0	0.0	0.0	0.0	0.0
Ranapur, District Jhabua	77	2.4 ± 0.1 (2.2 -2.7)	12.1 ± 0.3$^{\#}$ (11.5 -12.6)	1.3 ± 0.1 (1.2 - 1.4)	0.5 ± 0.1 (0.3 - 0.7)	0.9 ± 0.0$^{\wedge}$ (0.8 - 1.0)	3.3 ± 0.1 (3.1 - 3.6)	5.2 ± 0.3 (4.7 - 5.8)	1.0 ± 0.0 (0.9 - 1.1)	1.4 ± 0.1 (1.3 - 1.5)	1.0 ± 0.0	0.0 ± 0.0$^{\#}$ (0.0 - 0.0)	0.1 ± 0.0 (0.0 - 0.2)	0.0 ± 0.0 (0.0 - 0.1)	0.0	0.0 ± 0.0 (0.0 - 0.1)	0.0
Pushprajgarh, District Anuppur	52	2.5 ± 0.2 (2.2 -2.9)	12.3 ± 0.3 (11.8 -12.9)	1.4 ± 0.1$^{\wedge}$ (1.3 - 1.6)	0.4 ± 0.1 (0.2 - 0.5)	1.0 ± 0.1$^{\wedge}$ (0.9 - 1.2)	2.9 ± 0.1 (2.7 - 3.2)	5.7 ± 0.4$^{\wedge}$ (4.9 - 6.5)	1.1 ± 0.0 (1.0 - 1.2)	1.4 ± 0.1 (1.2 - 1.6)	1.0 ± 0.0	0.0	0.3 ± 0.1$^{\wedge}$ (0.1 - 0.4)	0.0	0.0	0.0	0.0
Malewada, District Gadchiroli	40	2.1 ± 0.2 (1.7 -2.4)	13.1 ± 0.3 (12.4 -13.7)	1.2 ± 0.1 (0.9 - 1.4	0.6 ± 0.2 (0.3 - 0.9)	0.7 ± 0.1 (0.5 - 0.9)	3.0 ± 0.2 (2.6 - 3.3)	4.2 ± 0.4 (3.4 - 4.9)	1.1 ± 0.1 (1.0 - 1.3)	1.4 ± 0.1 (1.2 - 1.6)	1.0 ± 0.0	0.0	0.1 ± 0.0 (0.0 - 0.1)	0.0 ± 0.0 (0.0 - 0.1)	0.0 ± 0.0 (0.0 - 0.1)	0.0	0.0 ± 0.0 (0.0 - 0.1)
Darekasa ,District Gondia	37	1.9 ± 0.2$^{\#}$ (1.6 -2.2)	13.1 ± 0.3 (12.4 - 13.7)	1.4 ± 0.1 (1.2 - 1.6)	0.2 ± 0.1$^{\#}$ (0.0 - 0.4)	0.8 ± 0.1 (0.7 - 1.0)	2.9 ± 0.2 (2.6 - 3.3)	4.4 ± 0.3 (3.7 - 5.1)	1.0 ± 0.0	1.5 ± 0.1 (1.3 - 1.7)	1.0 ± 0.0	0.0	0.0	0.0	0.3 ± 0.1$^{\wedge}$ (0.1 - 0.4)	0.0	0.0
Bekaria, District Udaipur	79	2.2 ± 0.1 (2.0 -2.5)	12.7 ± 0.3 (12.1 - 13.2)	1.4 ± 0.1 (1.2 - 1.5)	0.6 ± 0.1 (0.4 - 0.8)	0.8 ± 0.0 (0.7 - 0.9)	3.5 ± 0.1$^{\wedge}$ (3.2 - 3.7)	4.7 ± 0.2 (4.3 - 5.2)	1.3 ± 0.1$^{\wedge}$ (1.1 - 1.4)	1.2 ± 0.1$^{\#}$ (1.1 - 1.3)	1.0 ± 0.0	0.1 ± 0.1 (0.0 - 0.2)	0.0	0.0	0.0	0.0 ± 0.0 (0.0 - 0.1)	0.0
Devgadh Baria, District Dahod	34	2.4 ± 0.2 (2.0 -2.9)	12.2 ± 0.4 (11.4 - 12.9)	1.1 ± 0.1$^{\#}$ (1.0 - 1.3)	1.0 ± 0.2$^{\wedge}$ (0.6 - 1.3)	0.8 ± 0.1 (0.7 - 1.0)	2.7 ± 0.1$^{\#}$ (2.5 - 3.0)	4.2 ± 0.4 (3.3 - 5.0)	1.1 ± 0.1 (1.0 - 1.3)	1.3 ± 0.1 (1.0 - 1.5)	1.0 ± 0.0	0.2 ± 0.1 (0.0 - 0.5)	0.0	0.0	0.0	0.0 ± 0.0 (0.0 - 0.1)	0.0
Lavkar ,District Valsad	6	1.8 ± 0.3 (1.2 -2.4)	12.5 ± 0.8 (10.8 - 14.2)	1.5 ± 0.2 (1.1 - 1.9)	0.3 ± 0.3 (-0.3 - 1.0)	0.8 ± 0.2 (0.5 - 1.2)	3.2 ± 0.5 (2.2 - 4.1)	3.8 ± 1.2 (1.5 - 6.2)	1.3 ± 0.2 (0.9 - 1.7)	1.5 ± 0.2 (1.1 - 1.9)	1.0 ± 0.0	0.2 ± 0.2 (-0.2 - 0.5)	0.2 ± 0.2 (-0.2 - 0.5)	0.0	0.0	0.0	0.0
Manu bazar, South Tripura	27	2.5 ± 0.2 (2.0 -2.9)	12.6 ± 0.5 (11.6 -13.6)	1.3 ± 0.1 (1.2 -	0.1 ± 0.1$^{\#}$ (0.0 -	1.0 ± 0.1$^{\wedge}$ (0.9 -	2.6 ± 0.1$^{\#}$ (2.3 -	3.9 ± 0.4 (3.1 -	1.1 ± 0.1 (1.0 -	1.3 ± 0.1 (1.1 -	1.0 ± 0.0	0.1 ± 0.1 (0.0 -	0.0	0.0	0.1 ± 0.1 (0.0 -	0.0	0.0

				1.5)	0.2)	1.1)	2.8)	4.7)	1.3)	1.6)		0.2)			0.2)		
Santir bazar, South Tripura	39	2.4 ± 0.3 (1.9 -2.9)	13.2 ± 0.3 (12.6 -13.7)	1.3 ± 0.1 (1.1 - 1.4)	0.2 ± 0.1# (0.0 - 0.4)	0.7 ± 0.1 (0.6 - 0.9)	3.2 ± 0.2 (2.8 - 3.6)	3.9 ± 0.3# (3.3 - 4.6)	1.2 ± 0.1 (1.1 - 1.3)	1.3 ± 0.1 (1.1 - 1.5)	1.0 ± 0.0	0.1 ± 0.0 (0.0 - 0.2)	0.1 ± 0.0 (0.0 - 0.1)	0.0	0.1 ± 0.1 (0.0 - 0.2)	0.0	0.0

^ The mean number is significantly higher than Country Mean; # The mean number is significantly lower than Country Mean.

Table 3: The mean amino acid numbers and percentage of type 2 and type7 repeats shared in *Pf*HRP2 protein.

State	CHC	Amino Acid	Type 2		Type7		Type 2 + Type 7	
		Mean	Mean	%	Mean	%	Mean	%
Orissa	Bandhugaon	246.3	116.1	47.1	29.7	12.1	145.8	59.2
	Jagannathpur	239.2	117.4	49.1	21.5	9.0	138.9	58.1
Jharkhand	Jaldega	247.2	118.3	47.9	30.8	12.4	149.0	60.3
	Bano	245.4	118.6	48.4	28.4	11.6	147.0	59.9
Chhattisgarh	Jagdalpur	245.7	117.0	47.6	28.2	11.5	145.2	59.1
	Baikunthpur	244.4	129.6	53.0	20.4	8.3	150.0	61.4
Madhya Pradesh	Ranapur	242.2	108.5	44.8	31.2	12.9	139.7	57.7
	Pushprajgarh	250.6	111.1	44.3	34.3	13.7	145.4	58.0
Maharashtra	Malewada	237.2	117.7	49.6	24.9	10.5	142.6	60.1
	Darekasa	240.1	117.5	48.9	26.6	11.1	144.1	60.0
Rajasthan	Bekaria	242.9	114.2	47.0	28.5	11.7	142.6	58.7
Gujarat	Devgadh Baria	231.7	109.6	47.3	25.1	10.8	134.6	58.1
	Lavkar	237.5	112.5	47.4	23.0	9.7	135.5	57.1
Tripura	Manu bazar	235.3	113.3	48.2	23.3	9.9	136.7	58.1
	Santir bazar	240.7	118.4	49.2	23.7	9.8	142.1	59.0
**Overall**		**243.3**	**115.3**	**47.4**	**28.3**	**11.6**	**143.6**	**59.0**

Table 4: The percentage of various types of *Pf*HRP3 repeat present in eight different states of India.

State	CHC	n	Type1	Type2	Type4	Type7	Type15	Type16	Type17	Type18	Type20	Type28	Type29	Non Repeat Region
Orissa	Bandhugaon, District Koraput	114	100.0	0.0	100.0	100.0	100.0	98.2	100.0	98.2	100.0	0.9	0.0	100.0
	Jagannathpur, District Rayagada	7	100.0	0.0	100.0	100.0	100.0	100.0	100.0	85.7	100.0	0.0	0.0	100.0
Jharkhand	Jaldega, District Simdega	65	100.0	0.0	100.0	100.0	98.5	95.4	100.0	100.0	100.0	1.5	0.0	100.0
	Bano, District Simdega	8	100.0	0.0	100.0	100.0	100.0	100.0	100.0	100.0	100.0	0.0	0.0	100.0
Chhattisgarh	Jagdalpur	129	100.0	0.0	100.0	99.2	99.2	100.0	100.0	100.0	100.0	3.1	0.0	100.0
	Baikunthpur	6	100.0	0.0	100.0	100.0	100.0	100.0	100.0	100.0	100.0	0.0	0.0	100.0
Madhya Pradesh	Ranapur, District Jhabua	94	100.0	4.3	98.9	100.0	100.0	98.9	100.0	100.0	100.0	0.0	3.2	100.0
	Pushprajgarh, District Anuppur	67	100.0	0.0	100.0	100.0	100.0	100.0	100.0	100.0	100.0	6.0	3.0	100.0
Maharashtra	Malewada, District Gadchiroli	45	100.0	0.0	100.0	100.0	100.0	100.0	100.0	100.0	100.0	6.7	0.0	100.0
	Darekasa ,District Gondia	28	100.0	0.0	100.0	100.0	100.0	85.7	100.0	100.0	100.0	0.0	0.0	100.0
Rajasthan	Bekaria, District Udaipur	79	100.0	20.3	100.0	100.0	100.0	100.0	100.0	100.0	100.0	0.0	20.3	100.0
Gujarat	Devgadh Baria, District Dahod	53	100.0	3.8	100.0	100.0	100.0	94.3	100.0	100.0	100.0	0.0	3.8	100.0
	Lavkar ,District Valsad	4	100.0	0.0	100.0	100.0	100.0	100.0	100.0	100.0	100.0	0.0	0.0	100.0
Tripura	Manu bazar, South Tripura	14	100.0	0.0	100.0	100.0	100.0	92.9	100.0	100.0	100.0	0.0	0.0	100.0
	Santir bazar, South Tripura	37	100.0	0.0	100.0	100.0	100.0	100.0	100.0	100.0	100.0	5.4	0.0	100.0
**Overall**		**750**	**100.0**	**2.9**	**99.9**	**99.9**	**99.7**	**98.1**	**100.0**	**99.6**	**100.0**	**2.0**	**3.1**	**100.0**

Table 5: The average number of different type of repeats present in *Pf*HRP3.

CHC	n	Type1<br>Mean ± SD<br>(95%CI)	Type2<br>Mean ± SD<br>(95%CI)	Type4<br>Mean ± SD<br>(95%CI)	Type7<br>Mean ± SD<br>(95%CI)	Type15<br>Mean ± SD<br>(95%CI)	Type16<br>Mean ± SD<br>(95%CI)	Type17<br>Mean ± SD<br>(95%CI)	Type18<br>Mean ± SD<br>(95%CI)	Type20<br>Mean ± SD<br>(95%CI)	Type28<br>Mean ± SD<br>(95%CI)	Type29<br>Mean ± SD<br>(95%CI)	Non Repeat Region<br>Mean ± SD<br>(95%CI)
Bandhugaon,<br>District Koraput	114	1.22 ± 0.04<br>(1.13 - 1.31)	0.00	1.07 ± 0.03<br>(1.02 - 1.12)	1.18 ± 0.04<br>(1.11 - 1.25)	1.18 ± 0.04<br>(1.11 - 1.25)	8.82 ± 0.26<br>(8.31 - 9.34)	5.89 ± 0.14<br>(5.62 - 6.17)	2.23 ± 0.07<br>(2.10 - 2.36)	1.18 ± 0.04<br>(1.11 - 1.25)	0.01 ± 0.01<br>(-0.01 - 0.03)	0.00	1.18 ± 0.04<br>(1.11 - 1.25)
Jagannathpur,<br>District<br>Rayagada	7	1.00 ± 0.00	0.00	1.00 ± 0.00.	1.00 ± 0.00	1.00 ± 0.00	11.00 ± 0.76<br>(9.52 - 12.48)	6.57 ± 0.37<br>(5.85 - 7.30)	1.86 ± 0.34<br>(1.19 - 2.52)	1.00 ± 0.00	0.00	0.00	1.00 ± 0.00
Jaldega,<br>District<br>Simdega	65	1.14 ± 0.04<br>(1.05 - 1.22)	0.00	1.03 ± 0.02$^{\#}$<br>(0.99 - 1.07)	1.11 ± 0.04<br>(1.03 - 1.18)	1.09 ± 0.04<br>(1.01 - 1.18)	9.31 ± 0.36<br>(8.60 - 10.01)	5.89 ± 0.11<br>(5.67 - 6.12)	2.05 ± 0.07$^{\#}$<br>(1.91 - 2.19)	1.11 ± 0.04<br>(1.03 - 1.18)	0.02 ± 0.02<br>(-0.01 - 0.05)	0.00	1.11 ± 0.04<br>(1.03 - 1.18)
Bano,<br>District<br>Simdega	8	1.00 ± 0.00	0.00	1.00 ± 0.00	1.00 ± 0.00	1.00 ± 0.00	10.25 ± 0.37$^{\wedge}$<br>(9.53 - 10.97)	6.00 ±0.00	2.00 ± 0.00	1.00 ± 0.00	0.00	0.00	1.00 ± 0.00
Jagdalpur	129	1.11 ± 0.03$^{\#}$<br>(1.05 - 1.16)	0.00	1.12 ± 0.05<br>(1.03 - 1.22)	1.09 ± 0.03<br>(1.04 - 1.15)	1.08 ±0.03<br>(1.03 - 1.13)	9.29 ± 0.23<br>(8.83 - 9.74)	5.55 ±0.09$^{\#}$<br>(5.37 - 5.73)	2.18 ± 0.04$^{\#}$<br>(2.10 - 2.26)	1.10 ± 0.03<br>(1.05 - 1.15)	0.03 ± 0.02<br>(0.00 - 0.06)	0.00	1.10 ± 0.03$^{\#}$<br>(1.05 - 1.15)
Baikunthpur	6	1.17 ± 0.17<br>(0.84 -1.49)	0.00	1.00 ± 0.00	1.17 ± 0.17<br>(0.84 - 1.49)	1.17 ± 0.17<br>(0.84 - 1.49)	9.33 ± 1.31<br>(6.77 - 11.90)	5.67 ± 0.49<br>(4.70 - 6.64)	2.50 ± 0.50<br>(1.52 - 3.48)	1.17 ± 0.17<br>(0.84 - 1.49)	0.00	0.00	1.17 ± 0.17<br>(0.84 - 1.49)
Ranapur,<br>District Jhabua	94	1.15 ± 0.04<br>(1.08 - 1.22)	0.04 ± 0.02<br>(0.00 - 0.08)	1.07 ± 0.03<br>(1.01 - 1.14)	1.11 ± 0.03<br>(1.04 - 1.17)	1.12 ± 0.03<br>(1.05 - 1.18)	9.94 ± 0.28$^{\wedge}$<br>(9.39 - 10.49)	6.03 ± 0.15<br>(5.74 - 6.33)	2.60 ± 0.09<br>(2.42 - 2.77)	1.15 ± 0.04<br>(1.08 - 1.22)	0.00	0.03 ± 0.02<br>(0.00 - 0.07)	1.15 ± 0.04<br>(1.08 - 1.22)
Pushprajgarh,<br>District<br>Anuppur	67	1.04 ± 0.03$^{\#}$<br>(0.99 - 1.09)	0.00	1.04 ± 0.03<br>(0.98 - 1.11)	1.03 ± 0.02$^{\#}$<br>(0.99 -1.07)	1.03 ± 0.02$^{\#}$<br>(0.99 - 1.07)	9.28 ± 0.27<br>(8.75 - 9.82)	5.97 ± 0.10<br>(5.77 - 6.17)	2.07 ± 0.04$^{\#}$<br>(2.00 - 2.15)	1.03 ± 0.02$^{\#}$<br>(0.99 - 1.07)	0.06 ± 0.03<br>(0.00 - 0.12)	0.03 ± 0.02<br>(-0.01 - 0.07)	1.03 ± 0.02$^{\#}$<br>(0.99 - 1.07)
Malewada,<br>District<br>Gadchiroli	45	1.18 ± 0.06<br>(1.06 - 1.29)	0.00	1.04 ± 0.03<br>(0.98 - 1.11)	1.09 ± 0.04<br>(1.00 - 1.17)	1.04 ± 0.03$^{\#}$<br>(0.98 - 1.11)	9.71 ± 0.39<br>(8.95 - 10.47)	5.76 ± 0.13<br>(5.50 - 6.01)	2.13 ± 0.08$^{\#}$<br>(1.99 - 2.28)	1.09 ± 0.04<br>(1.00 - 1.17)	0.07 ± 0.04<br>(-0.01 - 0.14)	0.00	1.09 ± 0.04<br>(1.00 - 1.17)
Darekasa<br>,District Gondia	28	1.25 ± 0.08<br>(1.09 - 1.41)	0.00	1.39 ± 0.15<br>(1.10 - 1.68)	1.25 ± 0.08<br>(1.09 - 1.41)	1.25 ± 0.08<br>(1.09 - 1.41)	8.64 ± 0.81<br>(7.05 - 10.24)	5.43 ± 0.17$^{\#}$<br>(5.09 - 5.77)	2.21 ± 0.12<br>(1.98 - 2.45)	1.25 ± 0.08<br>(1.09 - 1.41)	0.00	0.00	1.25 ± 0.08<br>(1.09 - 1.41)
Bekaria,<br>District Udaipur	79	1.28 ± 0.05$^{\wedge}$<br>(1.18 - 1.38)	0.20 ± 0.05<br>(0.11 - 0.29)	1.06 ± 0.04<br>(0.99 - 1.14)	1.06 ± 0.03$^{\#}$<br>(1.01 - 1.12)	1.08 ± 0.03<br>(1.02 - 1.13)	8.29 ± 0.40$^{\#}$<br>(7.50 - 9.08)	5.62 ± 0.11<br>(5.40 - 5.84)	2.52 ± 0.10 $^{\wedge}$<br>(2.33 - 2.71)	1.27 ± 0.05$^{\#}$<br>(1.17 - 1.36)	0.00	0.20 ± 0.05$^{\wedge}$<br>(0.11 - 0.29)	1.28 ± 0.05$^{\wedge}$<br>(1.18 - 1.38)
Devgadh Baria,<br>District Dahod	53	1.15 ± 0.05<br>(1.05 - 1.25)	0.04 ± 0.03<br>(- 0.01 - 0.09)	1.04 ± 0.03$^{\#}$<br>(0.99 - 1.09)	1.11 ± 0.04<br>(1.03 - 1.20)	1.11 ± 0.04<br>(1.03 - 1.20)	9.68 ± 0.44<br>(8.81 - 10.55	6.32 ± 0.22$^{\wedge}$<br>(5.88 - 6.76)	2.68 ± 0.12$^{\wedge}$<br>(2.44 - 2.92)	1.15 ± 0.05<br>(1.05 - 1.25)	0.00	0.04 ± 0.03<br>(-0.01 - 0.09)	1.15 ± 0.05<br>(1.05 - 1.25)
Lavkar<br>,District Valsad	4	1.50 ± 0.29<br>(0.93 - 2.07)	0.00	1.25 ± 0.25<br>(0.76 - 1.74)	1.50 ± 0.29<br>(0.93 - 2.07)	1.50 ± 0.29<br>(0.93 - 2.07)	9.75 ± 1.93<br>(5.96 - 13.54)	6.75 ± 0.85<br>(5.07 - 8.43)	3.50 ± 0.29$^{\wedge}$<br>(2.93 - 4.07)	1.50 ± 0.29<br>(0.93 - 2.07)	0.00	0.00	1.50 ± 0.29<br>(0.93 - 2.07)
Manu bazar,<br>South Tripura	14	1.50 ± 0.14$^{\wedge}$<br>(1.23 - 1.77)	0.00	1.07 ± 0.07<br>(0.93 - 1.21)	1.50 ± 0.14$^{\wedge}$<br>(1.23 - 1.77)	1.50 ± 0.14$^{\wedge}$<br>(1.23 - 1.77)	7.21 ± 1.27<br>(4.72 - 9.71)	5.86 ± 0.54<br>(4.79 - 6.92)	2.57 ± 0.33<br>(1.93 - 3.21)	1.50 ± 0.14$^{\wedge}$<br>(1.23 - 1.77)	0.00	0.00	1.50 ± 0.14$^{\wedge}$<br>(1.23 - 1.77)
Santir bazar,<br>South Tripura	37	1.32 ± 0.10<br>(1.12 -1.53)	0.00	1.41 ± 0.14$^{\wedge}$<br>(1.13 - 1.68)	1.24 ± 0.07<br>(1.10 - 1.38)	1.24 ± 0.07<br>(1.10 - 1.38)	8.41 ± 0.65<br>(7.13 - 9.68)	5.84 ± 0.14<br>(5.56 - 6.12)	2.54 ± 0.11<br>(2.32 - 2.76)	1.24 ± 0.07<br>(1.10 - 1.38)	0.05 ± 0.04<br>(-0.02 - 0.13)	0.00	1.24 ± 0.07<br>(1.10 - 1.38)

$^{\wedge}$ The mean number is significantly higher than Country Mean; $^{\#}$ The mean number is significantly lower than Country Mean, ($p<0.05$).

Table 6: Limited polymorphisms in the non-repeat amino acid sequence of *Pf*HRP3 proteins in the Indian isolates.

Sample ID	Non-repeat amino acid sequence 1	Non-repeat amino acid sequence 2
**Reference sequence**	A N H G F H F N L H D N N S H T L H H A K A N A C F D D	A N H G F H F N L H D N N S H T L H H A K A N A C F D D
**Orissa**	A N H G F H F N L H **E** N N S H T L H H A K A N A C F D D	**Absent**
**Jharkhand**	A N H G F H F N L H D N N S H T L H H A K A N A C F D D	A N H G F H F **M** L H D N N S H T L H H A K A N A C F D D
**Chhattisgarh**	A N H G F H F N L H D N **D** S H T L H H A K A N A C F D D	A N H G F H F N L H D N **D** S H T L H H A K A N A C F D D
**Gujarat**	A N H **A** F H F N L H D N **D** S H T L H H A K A N A C F D D	A N H G F H F N L H D N N S H T L H H A K A N A C F D D

It is known that a majority of natural *P. falciparum* isolates carry a non-repetitive 28 amino acid sequence (in 3D7 laboratory isolates it is located at 171 codon to 198 codon) in the interior region of *Pf*HRP3 protein and this sequence is not found in *Pf*HRP2. However, some field isolates have been found to contain two non-repetitive 28 amino acid sequences (identical sequences) which are separated by histidine rich amino acid repeats. The reference sequence reported here indicates known non-repeat amino acid sequences of type 1 and type 2 as reported in Baker et al. 2010. Among 750 isolates with good quality sequences characterized in this study, only these 4 isolates had indicated amino acid variations.

Table 7: The average amino acid length and average number of type16 and 17 repeats in *Pf*HRP3 protein from different isolates.

State	CHC	Amino Acid	Type 16		Type17		Type 16 + Type 17	
		Mean	Mean	%	Mean	%	Mean	%
Orissa	Bandhugaon	167.3	52.9	31.7	29.5	17.6	82.4	49.3
	Jagannathpur	171.1	66.0	38.6	32.9	19.2	98.9	57.8
Jharkhand	Jaldega	165.5	55.8	33.8	29.5	17.8	85.3	51.6
	Bano	164.5	61.5	37.4	30.0	18.2	91.5	55.6
Chhattisgarh	Jagdalpur	163.6	55.7	34.1	27.8	17.0	83.5	51.0
	Baikunthpur	169.3	56.0	33.1	28.3	16.7	84.3	49.8
Madhya Pradesh	Ranapur	174.4	59.6	34.2	30.2	17.3	89.8	51.5
	Pushprajgarh	161.3	55.7	34.5	29.9	18.5	85.6	53.0
Maharashtra	Malewada	166.7	58.3	35.0	28.8	17.3	87.0	52.2
	Darekasa	168.5	51.9	30.8	27.1	16.1	79.0	46.9
Rajasthan	Bekaria	169.8	49.7	29.3	28.1	16.6	77.8	45.9
Gujarat	Devgadh Baria	174.8	58.1	33.2	31.6	18.1	89.7	51.3
	Lavkar	202.0	58.5	29.0	33.8	16.7	92.3	45.7
Tripura	Manu bazar	178.4	43.3	24.3	29.3	16.4	72.6	40.7
	Santir bazar	171.0	50.4	29.5	29.2	17.1	79.6	46.6
**Overall**		**168.3**	**55.0**	**32.7**	**29.2**	**17.4**	**84.2**	**50.1**
